# Contribution of forest wood products to negative emissions: historical comparative analysis from 1960 to 2015 in Norway, Sweden and Finland

**DOI:** 10.1186/s13021-018-0101-9

**Published:** 2018-09-04

**Authors:** Cristina-Maria Iordan, Xiangping Hu, Anders Arvesen, Pekka Kauppi, Francesco Cherubini

**Affiliations:** 1 Industrial Ecology Programme, Department of Energy and Process Engineering, Norwegian University of Science and Technology (NTNU), Trondheim, Norway; 2 Department of Environmental Sciences, University of Helsinki, Helsinki, Finland

**Keywords:** Negative CO_2_ emissions, Forest wood products, Carbon balance, Biomass, Forest management, Bioenergy, Life-cycle assessment

## Abstract

**Background:**

Forests and forest products can significantly contribute to climate change mitigation by stabilizing and even potentially decreasing the concentration of carbon dioxide (CO_2_) in the atmosphere. Harvested wood products (HWP) represent a common widespread and cost-efficient opportunity for negative emissions. After harvest, a significant fraction of the wood remains stored in HWPs for a period that can vary from some months to many decades, whereas atmospheric carbon (C) is immediately sequestered by vegetation re-growth. This temporal mismatch between oxidation of HWPs and C uptake by vegetation generates a net sink that lasts over time. The role of temporary carbon storage in forest products has been analysed and debated in the scientific literature, but detailed bottom-up studies mapping the fate of harvested materials and quantifying the associated emission profiles at national scales are rare. In this work, we quantify the net CO_2_ emissions and the temporary carbon storage in forest products in Norway, Sweden and Finland for the period 1960–2015, and investigate their correlation. We use a Chi square probability distribution to model the oxidation rate of C over time in HWPs, taking into consideration specific half-lives of each category of products. We model the forest regrowth and estimate the time-distributed C removal. We also integrate the specific HWP flows with an emission inventory database to quantify the associated life-cycle emissions of fossil CO_2_, CH_4_ and N_2_O.

**Results:**

We find that assuming an instantaneous oxidation of HWPs would overestimate emissions of about 1.18 billion t CO_2_ (cumulative values for the three countries over the period 1960–2015).We also find that about 40 years after 1960, the starting year of our analysis, are sufficient to detect signs of negative emissions. The total amount of net CO_2_ emissions achieved in 2015 are about − 3.8 million t CO_2_, − 27.9 t CO_2_ and − 43.6 t CO_2_ in Norway, Sweden, and Finland, respectively.

**Conclusion:**

We argue for a more explicit accounting of the actual emission rates from HWPs in carbon balance studies and climate impact analysis of forestry systems and products, and a more transparent inclusion of the potential of HWP as negative emissions in perspective studies and scenarios. Simply assuming that all harvested carbon is instantaneously oxidized can lead to large biases and ultimately overlook the benefits of negative emissions of HWPs.

**Electronic supplementary material:**

The online version of this article (10.1186/s13021-018-0101-9) contains supplementary material, which is available to authorized users.

## Background

Despite various international agreements and efforts to curb greenhouse gas (GHG) emissions, the continuing rise in anthropogenic emissions has led to unprecedented levels of atmospheric CO_2_ concentration, one of the dominant drivers of global warming [1]. In order to limit global warming to 2 °C above pre-industrial times, many emission scenarios from the Intergovernmental Panel on Climate Change (IPCC) rely on the removal of excess CO_2_ from the atmosphere with negative emissions [2–5]. Forest and forest products are important carbon sinks for the sequestration and storage of atmospheric carbon [6] and represent opportunities for negative emissions, essential in achieving long-term temperature stabilization targets [7–9]. Forests play an important role in the carbon cycle, removing about 2.4 Pg carbon per year from the atmosphere [10–12]. Global wood harvesting in 2011 amounted to 3 billion m^3^, accounting for 0.6% of the growing stock [13]. This roughly corresponds to 8 Gt CO_2_, of which about half were industrial round wood and half wood for energy [14].

After harvest, wood outtakes are usually not instantaneously oxidized, but remain stored as harvested wood products (HWP) for a period that varies from several months for bioenergy and paper, to many decades for timber used in buildings [15]. There is a time lag between emissions from harvested carbon (which can occur several decades after harvest) and sequestration of atmospheric carbon in re-growing vegetation (which starts right after harvest) [16]. The role played by the temporary storage of carbon in HWP is not insignificant [17]. The global pool of carbon stocked in HWPs was estimated to be 5 Gt in 2010, corresponding to 18.3 Gt CO_2_ [14], and is increasing at a rate of 150 Mt carbon per year, corresponding to 540 Mt CO_2_ [18]. Recent international accounting rules and scientific studies acknowledge the role of HWPs in national greenhouse-gas reporting [19–23]. For example, the recent EU legislation (EU 2018/841) requires the inclusion of the Land Use, Land Use Change, and Forestry sector (LULUCF) in the Paris Agreement goals, explicitly requesting member states to account for the climate mitigation potential of HWPs [24].

There are carbon balance studies that investigate the climate mitigation potential of HWPs and their role in net emissions from the forestry sector [19, 21–23, 25–27]. [28] estimate the current overall climate benefit from the Swedish forestry and HWPs use, in terms of reduced and avoided CO_2_ emissions, to be around 60 million t CO_2_ per year. They note a potential additional mitigation benefit of 40 million t CO_2_ per year under a scenario with a 50% increase of the sustainable harvested biomass. [29] investigate the climate change potential of two HWP strategies for the Canadian forest sector using a carbon model based on the Tier 3 approach from the 2013 IPCC [30], and find that up to a cumulative mitigation potential of 254 Tg CO_2_ in 2030 and 1180 Tg CO_2_ in 2050 can be achieved. [31] estimates the net-emissions from wood products for each of the 26 EU countries using production and trade data, following the methodology proposed in the 2006 IPCC Guidelines [32] for the delayed emissions, and estimate net-emissions from the HWP pool of − 4 million t CO_2_ per year in Finland and − 3.9 million t CO_2_ per year in Sweden for the historical period 1990–2009. [25] use the Tier 2 method proposed in the 2013 IPCC [30] to estimate emissions and removals from the HWPs from 1990–2030 in EU with three future harvest scenarios (constant historical average, and ± 20% in 2030). They quantify the HWP sink at − 44.0 Mt CO_2_ per year for the historical period 2000–2012 and forecast a decreasing trend until 2030 (− 22.9 Mt CO_2_ per year). [26] show that the average amount of C in the European HWP pool is equal to 1843 Tg C for the period 2000–2012. In addition, they estimate that in 2030 the carbon stock changes in the EU forest pools (including HWPs) would reach a sink of 126 Tg C per year under the scenario assuming constant harvest and afforestation rates as in the aforementioned historical period.

Far backward-looking historical analyses of HWP sectors at a country level with a quantification of their potential role for negative emissions are relatively rare, while climate impact studies that simply assume instantaneous oxidation of forest wood outtakes (thereby ignoring HWPs and delayed emissions) are relatively frequent [33–37]. There are approaches to incorporate the dynamic nature of carbon flows in forest production systems and in the HWP’s life-cycle, explicitly taking into account the timing of carbon emission [38–41]. These include modelling decay rates of HWPs using an exponential function based on the half-lives of the products [30], or more realistic probability distribution centred around the mean half-life of the product, for example the gamma distribution [15, 17] or the chi distribution [42, 43]. These approaches facilitate the quantification of climate change impacts of HWPs using life-cycle assessment (LCA), a commonly used tool for estimating the environmental performance of products or processes through their entire value chain [44].

In this paper, we perform a bottom-up analysis to compare the forestry sector for the period 1960–2015 in Norway, Sweden, and Finland, three countries with advanced forestry industries, and estimate the corresponding historical net CO_2_ emissions. First, we provide the yearly outtake volumes per species of tree and wood class and map the annual manufacturing of HWPs, grouped by specific product categories on the basis of their half-lives. Then, we estimate the CO_2_ emission rates from the temporal decay of HWPs over time and estimate the corresponding CO_2_ removal from the atmosphere from forest regrowth. We quantify the CO_2_ storage over time in HWPs and the time-distributed decay of HWPs, and thereby estimate the net CO_2_ emissions as the difference between the CO_2_ oxidizing from HWPs and the time-distributed CO_2_ removals by vegetation regrowth. Finally, we integrate the historical trends in HWP production with the EcoInvent LCA database in order to produce carbon footprints of the forest-based products.

## Methods

### Study areas

The geographical scope of this study is the region of Northern Europe, which includes Norway, Sweden and Finland. Managed forests in this region are dominated by Norway spruce (*Picea abies),* Scots Pine (*Pinus sylvestris)* and Birch (silver birch—*Betula pendula,* and downy birch—*Betula pubescens).* The region experiences a boreal climate characterized by long cold winters with short mild summers and moderate, seasonally-distributed precipitation [45]. Scandinavian managed forests are long-rotation biomass production systems, enabling continuous wood harvests whilst sustaining constant and even growing standing stocks.

In Norway, forest covers 33% of the country’s land area, equating to almost 10 million ha. The total growing stock in Norwegian forests is 952 million m^3^, of which 849 million m^3^ belong to the productive forest area. The stock is steadily increasing and, over the last 10 years, the volume has grown 25%. The annual increment in 2016 was 25.8 million m^3^, with 23.8 million m^3^ in productive forest, of which 18.5 million m^3^ is conifer [46]. Commercial round wood removals are around 10 million m^3^ each year. Spruce is the most common species of tree (44% of the total growing stock), followed by pine (31%) and broad-leaf (25%). The presence of broad-leaved species is increasing; over the last ten years the volume of broad-leaved species has increased by 40% [47].

In Sweden, productive forests cover 57% of the total Swedish land area or 23 million ha. The total growing stock in the Swedish forest is just over 3.3 billion m^3^, a 98% increase since the mid 1920’s. In the past 90 years, Sweden’s forest assets have doubled [48]. The annual increment stands at around 120 million m^3^, and 97 million m^3^ of that growth was harvested in 2015 [49]. The composition of Sweden’s forest is coniferous forest (83%), mixed forest (12%) and pure deciduous forest (5%). The volume of wood comprises 42% spruce, followed by 39% pine, 12% birch, and 7% other deciduous trees [50].

In Finland, forest covers 75% of the country’s land area or almost 22.8 million ha [51]. The total volume of growing stock in Finland’s forests is almost 2.4 billion m^3^ and has increased more than 40% during the last 40 years. Biomass removals from Finnish forests have increased considerably over the last few decades. In 2015, the annual increment of growing stock was 105.5 million m^3^ while 68 million m^3^ were harvested [52]. Almost 50% of the volume of the timber stock consists of pine, 30% of spruce and 20% of broadleaved species [53].

### Historical data collection

We gather and process data for the harvested amounts of wood per species (spruce, pine and birch) and per class (saw log, pulpwood and wood for energy) for the period 1960–2015 in Norway, Sweden, and Finland. For each country, we use national statistics services and publicly reported data from FAO Forestry for the HWP and bioenergy production to quantify final product volumes. We do not consider exports nor imports. Final products are aggregated into the following categories (based on their average lives in the anthroposphere as reported in [16]): paper (4 years), packaging items (9 years), furniture and building maintenance (43 years), and buildings (140 years). Bioenergy represents a by-product of the forest sector, as a large share of the wood-based energy is produced as a side product from forestry and only small shares from independent production. For bioenergy production, we use the following categories: district heating, and energy consumption in households with three different technologies: wood stoves (new and old), open fire, and self-produced bioenergy within industry. We also perform a statistical analysis to assess the variability of the data with respect to the historical mean trends, and identify potential outliers for each combination of wood species and class. We use a historical fit to see the sensitivity of the wood harvest rates to random fluctuations, for example market-related shifts. In addition, we estimate for each country the annual incremental rate of change to facilitate interpreting the national trends. We also provide the covariance coefficients of the historical trends within each country (between the wood species and classes) and between the countries.

#### Norway

For the harvested amounts of wood we use the data reported by Norwegian National Statistics from 1960 to 2015 [47]. We fill the 1960–1979 data gaps on harvested pine and spruce species by assuming the same shares as for the interval 1980–1989. For the wood for energy removals we use the data reported by FAO Forestry [54]. Final products are estimated by aggregating data for production of pulp and paper reported by the Norwegian Pulp and Paper Association [55] with those from construction timber production from Treindustrien [56] and production data from FAO Forestry for other HWP categories. For bioenergy, we use data reported by Norwegian National Statistics and we complete missing data with assumptions (see Additional file 1: Table S1). We do not take into consideration the harvested amounts of wood for energy for private use, since such figures are not reported by Norwegian National Statistics. Wood stoves produced between 1970 and 1998 are categorized as old stoves and had few or no glass windows. Such stoves also had no wall air flushing, insulation of combustion chamber, or secondary air. Almost all stoves produced after 1998 (after new regulations setting stove particle emissions limits were adopted) exhibit new technology, e.g. secondary air, glass flushing, insulated combustion chamber and sometimes double glazing.

#### Sweden

We use the national official statistics for the harvested data reported by the Swedish Forest Agency [50] and we complete missing information with data from FAO Forestry. The data for HWP are from the Swedish Forest Industries Federation [57], complemented with data from FAO Forestry [54]. For bioenergy data we use Statistics Sweden data [58], but no data are available for the period 1960–1989. We thus assume the same share of consumption of wood fuels for each category (district heating, households, industry, electricity production) as 1990 levels. For more details, see Additional file 1: Table S2.

#### Finland

We use the national Official Statistics of Finland [51] for the data on harvested amounts. We collect statistics for each year between 1960 and 2015 from both LUKE [51] and FAO [54] for the flows of HWP and bioenergy production in Finland (see Additional file 1: Table S3 for a full list of references).

### Decay and regrowth functions

Each HWP category has a particular mean life in the anthroposphere before the carbon stored in products is released back to the atmosphere. We define the following product categories: bioenergy, paper, packaging items, pulp, furniture and building maintenance, and buildings. We use the half-lives of the products provided in Guest and Strømman [59] as the mean of the Chi square distribution to model the oxidation rate of wooden products over time (see Additional file 1: Table S4). Other approaches are used in the scientific community, such as an exponential decay function or a gamma distribution [15, 17]. An exponential decay rate can be an over-simplification of the real decay rate, as the decay peak will likely occur around the mean life of the products rather than immediately after harvest. In contrast, the gamma distribution has a more realistic oxidation profile, but it requires two parameters to be specified (i.e., mean half-life and year of expected 95% oxidation) [17]. The Chi square distribution is a special case of the gamma distribution, as it only requires the mean half-life of the product to shape the bell-like decay curve [42]. We therefore produce one Chi square distribution per product category and then make a convolution with the amount of carbon in each category for the period 1960–2015. This gives the actual profile of CO_2_ emissions from the oxidation of HWPs.

In order to estimate negative emissions, a set of simplified assumptions are needed to schematically model forest regrowth. We assume that once the wood is harvested, the same species are replanted and they start sequestering CO_2_ from the atmosphere, and the same amount of carbon harvested will be sequestered during regrowth by the end of the rotation period. For the emission profiles of the bioenergy we use the data of final consumption, which might differ from the data of outtake volumes owing to unreported logging and cascading uses of biomass (e.g., bioenergy from industrial residues). We use another Chi square distribution to schematically model the sequestration of atmospheric CO_2_ in the growing forest, which is calibrated on the specific rotation period of each tree species (see Additional file 1: Table S5). We then model the CO_2_ uptake profiles as a convolution between the wood outtakes from 1960 to 2015 for each species of tree and the respective Chi square distribution. For simplicity, we do not consider the oxidation of the wood residues left after harvest on the forest floor.

The net CO_2_ emission profiles are finally estimated by summing the time distributed CO_2_ oxidation flows from the HWPs and the CO_2_ removal flows from the forest regrowth.

### Integration with the emission inventory database

We quantify emissions of the three most important GHGs, fossil CO_2_, fossil CH_4_ and N_2_O, by linking the annual quantities of HWP categories produced in each country with the product-specific life-cycle emission factors from the EcoInvent 3.2 database [60]. This information can be instrumental to future LCAs of HWP systems and can facilitate the consideration of these emissions in climate impact analysis of the forestry sector. We adapt the emission inventories for each country by taking into consideration the national electricity mixes. Owing to a lack of data on historical emission factors, we use fixed emission factors. For the emission factors for fibreboards, plywood and particleboards we calculate the average between products manufactured with raw wood and products where residual wood fractions are used. For bioenergy production, in order to avoid double counting, we calculate the emissions based on the wood for energy outtakes data reported by the national statistics. Emission factors for residential wood stoves are obtained from [61]. We use the data from [61] representing old stoves (manufactured before 1998) and new stoves (manufactured after 1998) with partial load operation. For open fires, we use the emission factors from indirect emissions from stoves. For Sweden’s and Finland’s residential wood burning, we use an average of the three types of technologies (old stoves, new stoves, open fireplaces). In Norway, we only look at the emissions from households, since most of the inputs for the district heating sector in Norway are by-products of other wood industries. In Sweden and Finland, we use the EcoInvent 3.2 data for heat production and electricity.

## Results and discussion

### Historical wood outtakes

**Fig. 1:**
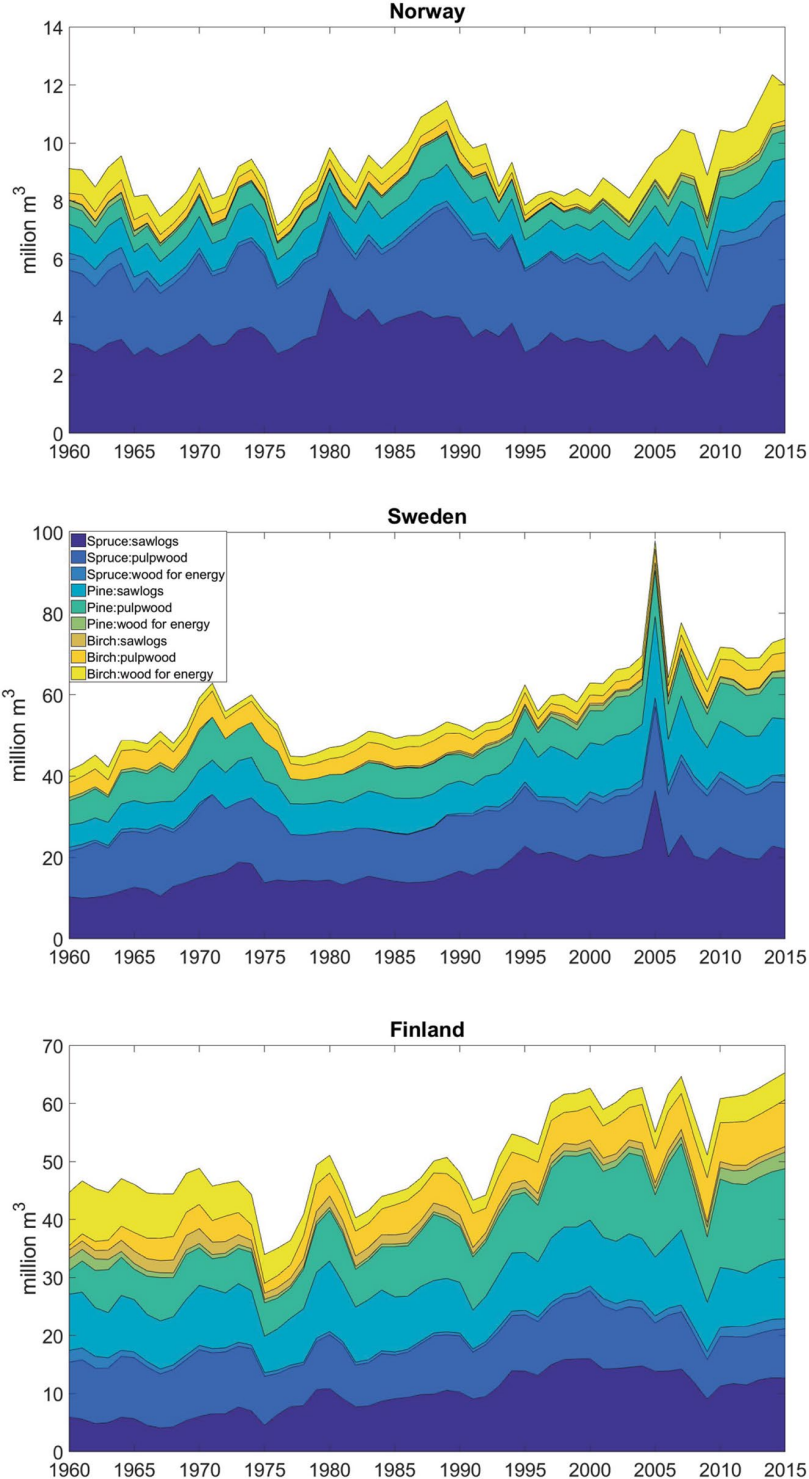
Historical wood harvest outtakes in Norway, Sweden and Finland between 1960 and 2015 with a breakdown on tree species (Spruce, Pine and Birch) and wood classes (saw logs, pulpwood and wood for energy)

Figure 1 shows the different historical trends in forest wood outtakes in the three countries. We estimate that a total of approximately 6.6 billion m^3^ of wood were harvested between 1960 and 2015 in the 3 countries, and 638 million m^3^ (less than 10%) of this volume were harvested for direct energy production. The largest growth in annual harvested amounts of wood is in Sweden where, relative to 1960, volume outtakes in 2015 increase by 79%, followed by Finland with 46%. In Norway, the annual wood outtakes increased by about one-third, from approximately 9 million m^3^ in 1960 to almost 12 million m^3^ in 2015. Spruce as saw logs and pulpwood cover the largest shares of the harvested volumes in Norway (around 58% on average for the 1960-2015 period), while birch is the dominant species for bioenergy use. In Sweden, spruce as saw logs and pulpwood represents the largest outtakes (almost 56% of the total), followed by pine saw logs and pulpwood, which on average represent 16% and 14% of the total harvested amounts. In Finland, with almost equal average shares of 19% each, the largest volumes harvested are spruce as saw logs and pulpwood and pine as saw logs and pulpwood, and birch as pulpwood and wood for energy have equal shares of 9%. The data for pine pulpwood show a historical increase, from 9% in 1960 to almost 23% of the total outtake in 2015.

The historical trends show some similarities in the forest industries of these countries. The effects of the oil crisis from the early 1970s are visible in all three countries, where there is a sudden drop in the harvested amounts at the beginning of the decade. Similarly, the financial crisis from 2008 is the reason for another decline in the total amounts of wood outtakes in all three countries. The 2005 peak in Sweden, and the following sudden drop in Finland, represents the markets’ response to the effects of the Gudrun storm in January 2005, when more than 75 million m^3^ of trees were destroyed in southern Sweden. This resulted in Sweden experiencing the world’s largest surplus of lumber to date, and a 41% increase in harvested amounts from the previous year. Therefore, Finland registers a 12% decrease from the 2007 level, due to a market distortion from Sweden. The trends in the total harvested volumes for Sweden and Finland are in line with the ones reported by [31] for the period 2000–2009.

The use of bioenergy in the different countries depends on the historical context and on the national systems for district heating and electricity production. District heating systems are often seen as an infrastructure that could facilitate the transition to low-carbon energy systems. Finland and Sweden are very dependent on their forest industries and account for the highest use of biomass for energy purposes on a per capita basis among high income countries [62].

Norway is self-sufficient in energy, with domestic energy consumption being dominated by electricity, mainly derived from hydropower (96%) [47]. Consequently, bioenergy holds a small share (6%) of domestic energy consumption, of which domestic users use approximately 50% for heat production with small wood-burning stoves. In Norway, about 53% of the domestic consumption of wood biofuels for heat production is used in households, 24% in the pulp and paper production, 11% as wood chips and bark in central district heating, 3% as briquettes and pellets, and the remaining 9% in other industries, including sawmilling [63].

In the 1960s, most Swedish buildings used fuel oil to cover their heating demands, while district heating accounted for only 3% of the heat market. Today district heating is the dominant source of heating and accounts for 58% of the energy purchased for heating of buildings, while fuel oil accounts for less than 2% [64]. The harvested stem wood is mainly used by the pulp, paper and sawmill industries. Substantial residue flows in the industry are primarily used internally as process energy, but also delivered to the external energy market such as district heating. In 2013, bioenergy contributed 23% of the total primary energy supply (470 PJ), with about 85% coming from the forest (logging and forest industrial residues) [65].

Finland uses subsidies to support bioenergy generation in order to achieve policy targets for renewable energy. Wood-based fuels cover 88% of the total renewable energy generation [66]. If harvesting levels remain the same, moving biomass from energy use to material use implies that the local energy supply will decrease in the short term, which can hinder achieving local renewable energy targets [67]. In 2015, 7.35 million m^3^ of wood chips, 10.1 million m^3^ of industrial by-products and 0.68 million m^3^ of recycled wood were combusted [68]. In the Finnish energy supply wood fuels, especially black liquor, also have a large role, accounting for one-fifth of the annual total energy consumption [51]. The local use of wood chips for energy is expected to increase by about 64 PJ (9 Mm^3^)–86 PJ (12 Mm^3^) in 2020 [67].

### Statistical analysis of historical wood outtakes

**Fig. 2:**
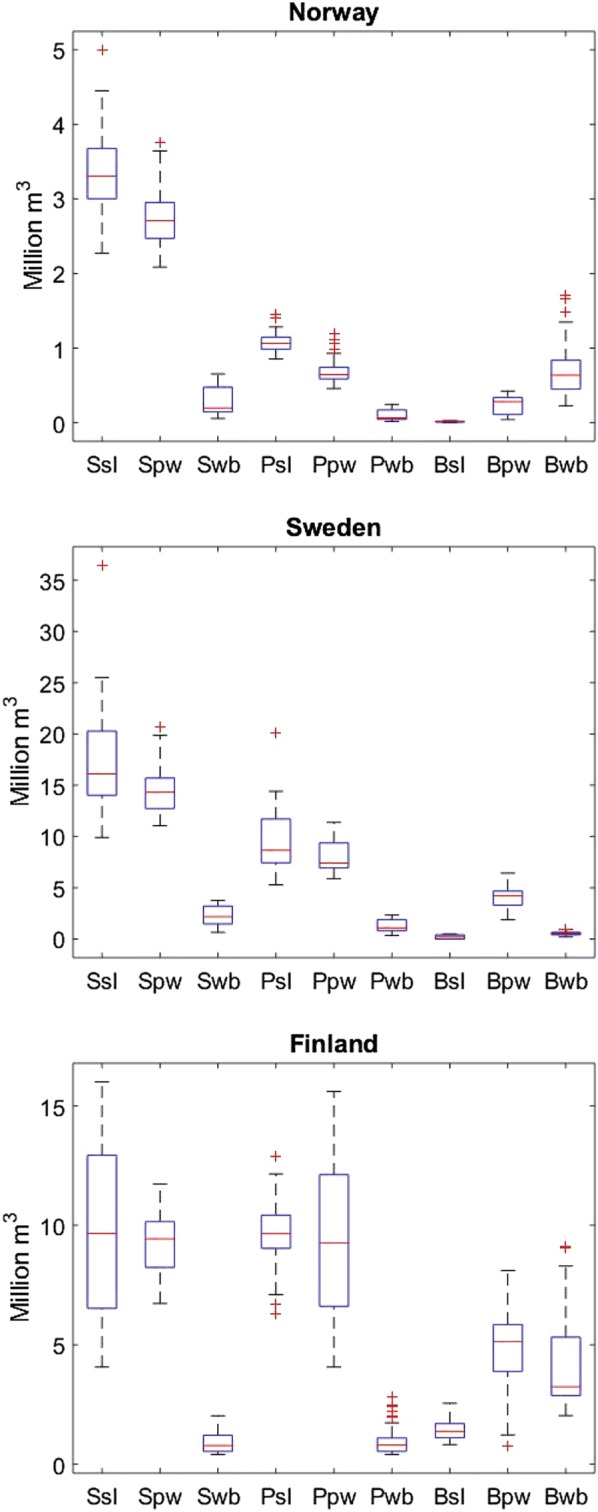
Boxplot of the wood harvest data in Norway, Sweden and Finland for the different tree species and wood classes. The first upper-case letters stand for the tree species with S for spruce, P for pine and B for birch, while the second and third letter stand for wood classes with sl for sawlogs, pw for pulpwood and wb for wood for bioenergy. The central red line in each box indicates the median, and the bottom and top edges of the box are the 25th and 75th percentiles, respectively. The whisker indicates 1.5 times of the 75th percentile minus the 25th percentile to the bottom or top edge of the box if outliers are detected. Otherwise, it indicates the minimum (bottom whisker) or the maximum (top whisker). The outliers are the points that fall outside of the whisker and are indicated with the red ‘ + ’ symbol

Figure 2 shows an overview of the data distribution of the historical datasets using a statistical analysis based on boxplots. We perform this statistical analysis to test the variability of the trends in the harvesting amounts in the three respective countries. This analysis provides an idea of which wood species and classes are more stable or, conversely, more sensitive to inter-annual variations driven by market dynamics or changes in national conditions. This analysis can also inform whether the species and classes usually respond with an increase (positive outlier) or decrease (negative outlier) in outtake volumes to disturbances. The outliers are the points that fall outside of the whisker and are indicated with the red ‘ + ’ symbol. The whisker indicates 1.5 times of the 75th percentile minus the 25th percentile to the bottom or top edge of the box if outliers are detected. Otherwise, it indicates the minimum (bottom whisker) or the maximum (top whisker). By far, the largest number of outliers are in historical wood outtakes in Norway and Finland, especially for the categories spruce saw logs, pulpwood and birch wood for energy which demonstrate large inter-annual variation. Norway has only positive outliers, whereas Finland also has a few negative outliers (pine saw logs and birch pulpwood). The trend is much more stable in Sweden, with only a few outliers for spruce saw logs; pulpwood and pine saw logs due to the storm in 2005. This means that the Swedish forest sector is more stable and more independent of the financial market dynamics than the Norwegian and Finnish ones, which are more sensitive to external conditions.

In addition, we estimate the annual incremental rate for each country and we find that for the historical period 1960–2015, Sweden has the highest annual increase of 0.48 million m^3^ per year, followed by Finland with 0.40 million m^3^ per year and respectively Norway with 0.03 million m^3^ per year (for more details please see Additional file 1: Table S7).

We also perform a correlation analysis and estimate the covariance coefficients of the total national historical harvested volumes (see Additional file 1: Table S8–S9). There is a strong positive correlation between total harvest volumes in Sweden and Finland (0.72), while Norway is less positively correlated with the other two countries (0.33 with Sweden and 0.38 with Finland). This means that harvest volumes in Sweden and Finland have been historically coupled.

When extending the statistical analysis and looking at the covariance coefficients between countries at the tree species level, the strongest correlation is found between the pine harvested volumes in Sweden and Finland (0.79). Correlation coefficients are usually smaller with Norway (see Additional file 1: Table S10).

**Fig. 3:**
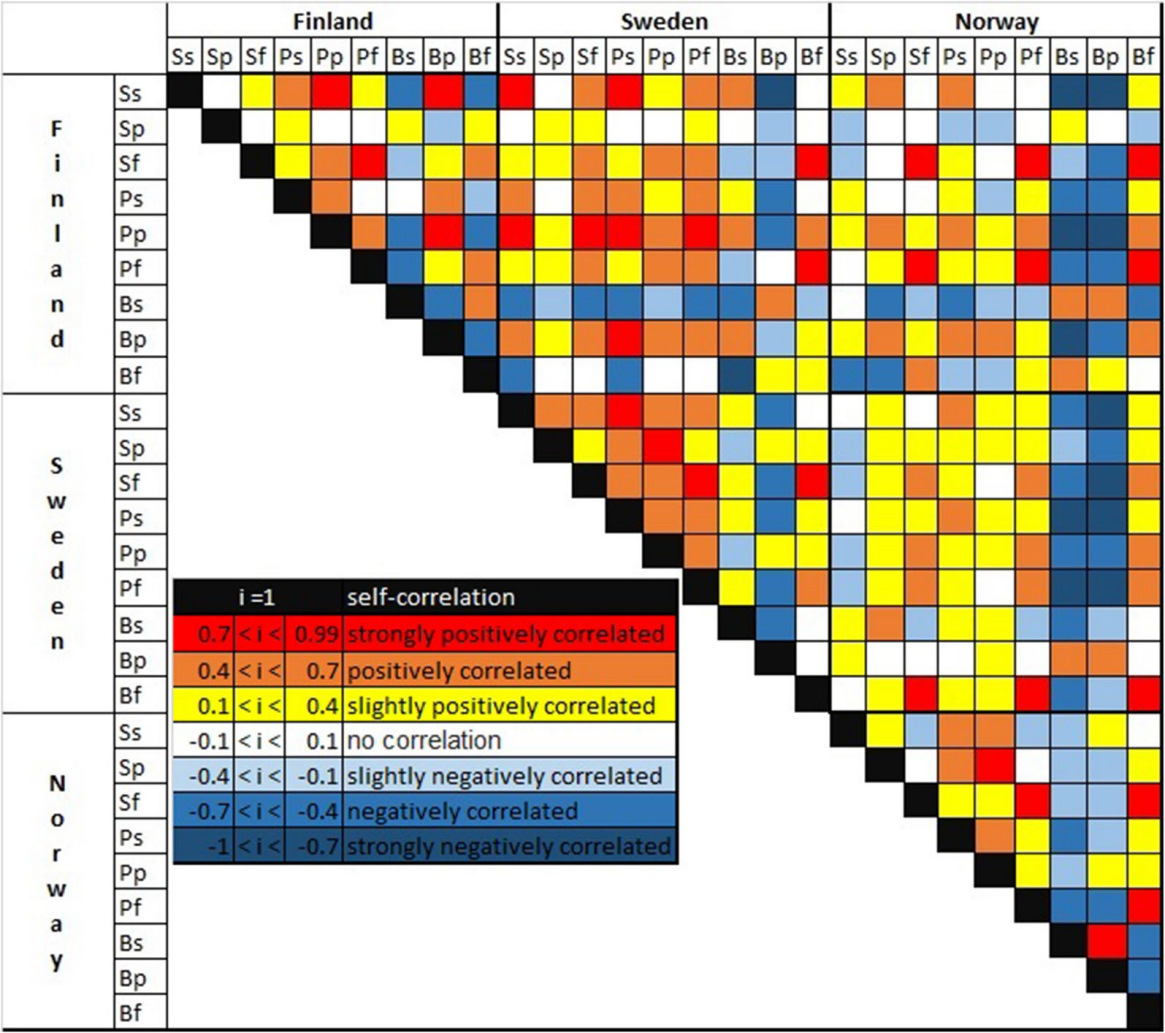
Heat map of the covariance coefficients of the historical trends of harvested volumes within each country (diagonal) and between each two countries, with a breakdown on the tree species and class, where the first capital letter indicates the tree species (S: Spruce; P: Pine; B: Birch) while the second letter indicates the wood class (s: saw logs; p: pulpwood; e: wood for energy)

In Fig. 3, we present a heat map of the covariance coefficients at the wood classes’ level between each two countries and within each country (on the diagonal). Our results generally reveal significant positive correlations for the wood for energy harvesting volumes between each two pair of countries. This relation suggests similar increasing trends in the production of energy from wood sources across the region. For example, we find high covariance coefficients between birch harvested for energy in Sweden and Norway (0.75), spruce for energy in Finland and birch for energy in Norway (0.70), spruce for energy in Finland and birch for energy in Sweden (0.80). In addition, we find very strong positive correlation between spruce saw logs in Finland and Sweden (0.76) which highlights the sustained growth (during the time period 1960–2015) of the harvested amounts for this species and class as we already graphically presented in Fig. 1. The correlation is more random and/or negative for birch classes, indicating that harvest volumes of birch are essentially decoupled. The statistical analysis within each country’s trends also reveals negative correlation between birch for energy and birch saw logs and pulpwood in the case of Norway, which indicates a possible interchangeability between them. Same relationship is indicated in the case of Sweden between birch pulp wood and spruce and pine saw logs, in the case of Finland between birch saw logs and spruce saw logs. More detailed information, with values of the correlation coefficients, are available in the Additional file 1: Tables S11–S16.

### Historical production of HWP

**Fig. 4:**
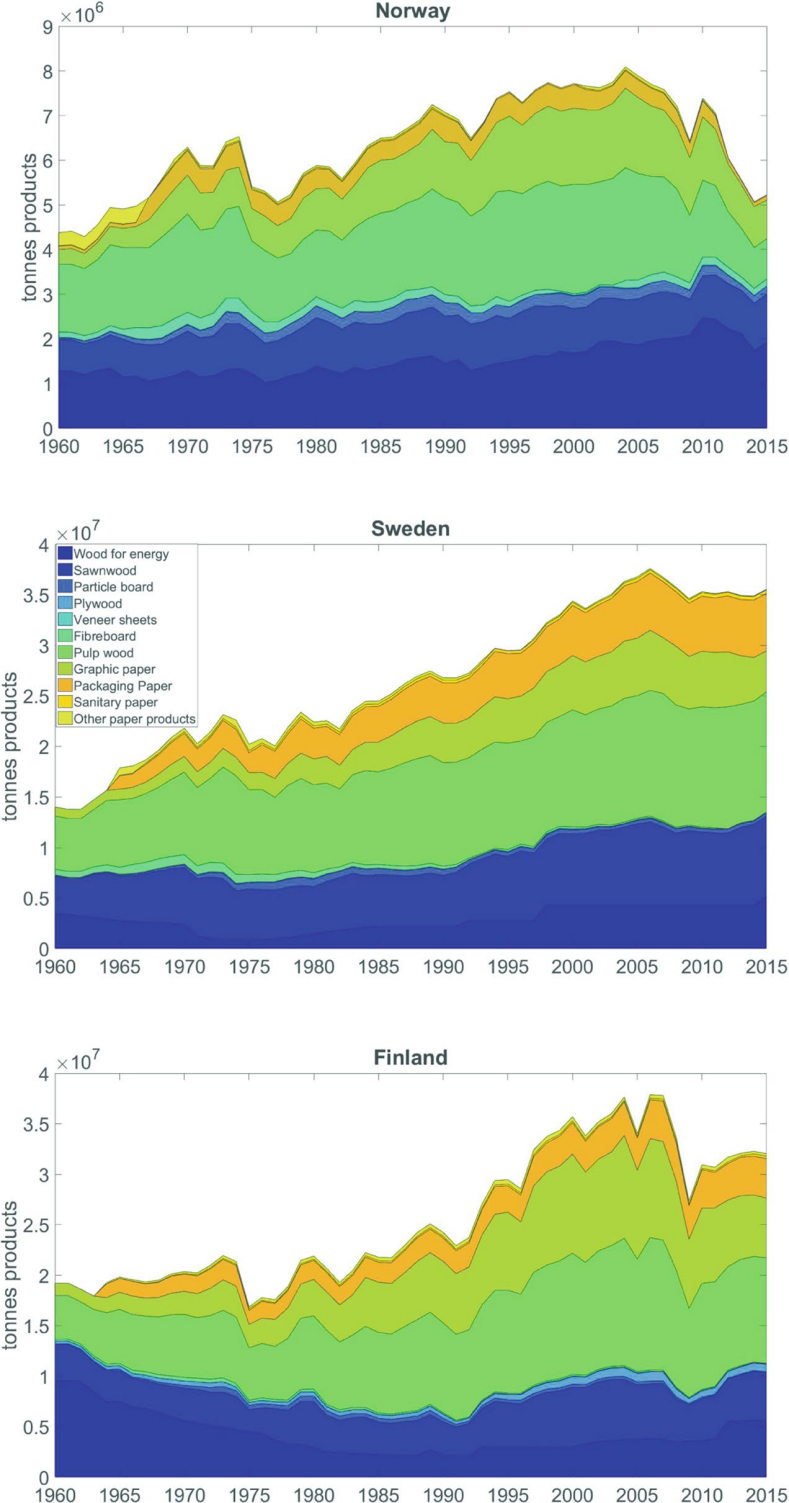
Fate of harvested wood in Norway, Sweden and Finland between 1960 and 2015 with a breakdown on product categories: wood for energy, sawn wood, particle board, plywood, veneer sheets, fibreboard, pulp wood, graphic paper, packaging paper, sanitary paper and other paper products

Figure 4 shows the annual production of final HWPs from 1960 to 2015 in each of the three countries. In total, 2.3 billion tonnes of solid products were manufactured from the three countries. In Norway, there is an increasing trend in bioenergy production, peaking in 2010. This is a consequence of the ‘Strategy for increased expansion of bioenergy’ adopted in 2008 [69], which aims at increased use of bioenergy for heating, as well as at an increase in the supply of forest based fuels. On the other hand, fibreboard and paper production has decreased since 2009, although the harvesting amounts have been increasing since 2004 (see Fig. 1). This is due to the increased exports of industrial round wood (from ca. 0.5 million m^3^ in 2005 to nearly 4 million m^3^ in 2015), which is the cause for the sharp decline in total domestic products after 2010.

Sweden’s pulp production (including mechanical, semi-mechanical, chemical and semi-chemical pulp) has expanded by 120%, from 5.2 million tonnes at the beginning of the 1960s to 11.6 million tonnes in 2015. The paper production has also expanded, with an 11-fold increase, from 0.9 million tonnes at the beginning of the period to about 10 million tonnes in 2015. In addition to the market demand, this expansion can also be explained by changes in the technological process; today the modern paper production involves the addition of other raw materials, for example kaolin. Approximately 45% of the harvested wood in Sweden, corresponding to around 40 million m^3^, becomes timber. This, in turn, becomes furniture, construction timber (boards and planks) and other wood products. Another 40–45% of the harvested wood, corresponding to around 35 million m^3^, becomes pulpwood, newspapers, packaging and other paper products, while just under 10% (8–9 million m^3^) becomes biofuel, which is mainly used for electricity and heating.

The amounts of wood for energy production in Sweden have steadily increased since the 1970s. This growth occurred simultaneously with the expansion of the district heating network during the 1960s and 1970s, facilitated by the high rate of housing construction stimulated by the million homes programme (1965–1974) [62]. The second sharp increase at the beginning of the 1990s can be interpreted as a consequence of the energy tax reform in 1991, when the cost of coal in production of district heat dramatically increased, thereby making biomass more convenient [62]. The increasing trend from 2015 is probably an effect of the scheme for Tradable Renewable Electricity Certificates (TRECs) and of the joint market for TRECs between Sweden and Norway. Today, bioenergy is the leading energy source in Sweden, increasing from 40 TWh per year in the 1970s to around 140 TWh per year in 2014. In 2009, bioenergy surpassed oil as the leading energy source for Swedish energy consumption. Today, district heating satisfies about 60% of the heat demand in Swedish buildings [62].

In Finland, there is a sharp increase in the paper industry from the middle of the 1990s until 2008, when the production volumes start decreasing due to the financial crisis and the move towards digital communication media. Timber production shares a similar trend with an accelerated growth after 1990, a sudden drop in 2008, followed by a further increase, however not reaching the full potential from before. The share of Finnish HWPs of the value of total exports was the largest in the world in 2013, namely 20%, mainly due to the pulp and paper industries [51]. Currently, the Finnish production of sawn softwood represents almost 3% of the total global production, while wood pulp accounts for 6%, and paper and paperboard for 3% [51].

### Emission profiles from oxidation of HWPs

**Fig. 5:**
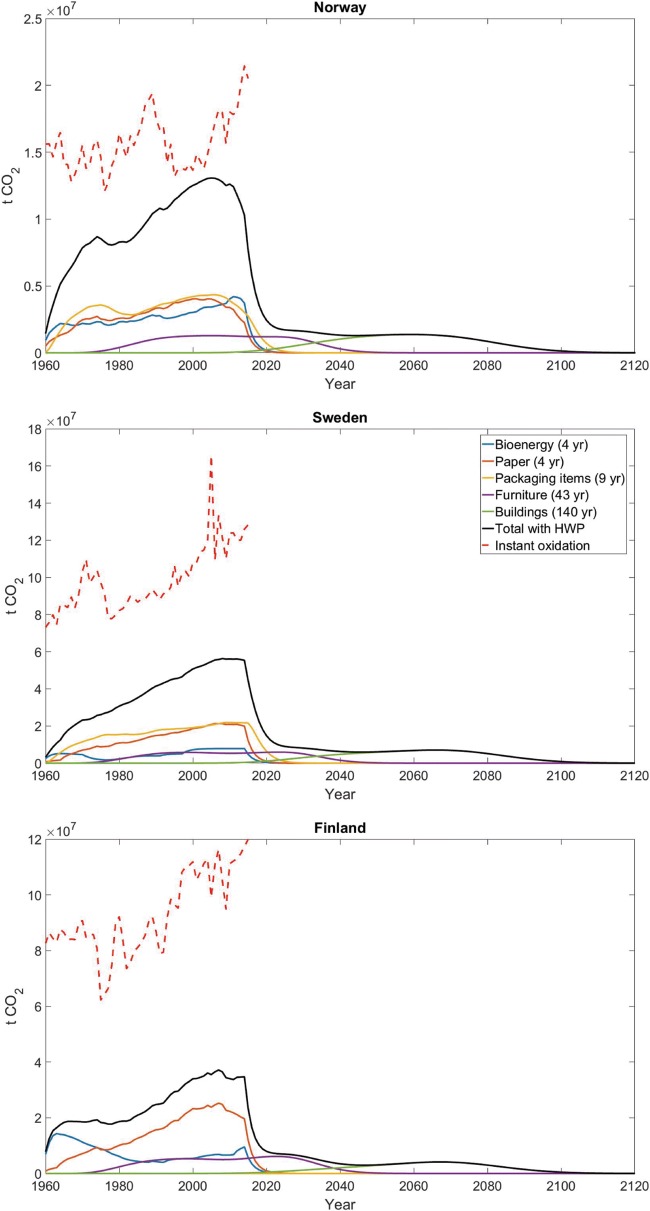
CO_2_ emission profiles in Norway, Sweden and Finland with breakdown on emissions from individual final product categories (bioenergy, paper, packaging items, furniture, and buildings) and national total (black). The potential emission if instantaneous oxidation is assumed immediately after harvest (red dotted line) is also shown as a benchmark

Figure 5 shows the CO_2_ emission profiles from oxidation of HWPs in each country, based on the product-specific mean half-life and Chi square distribution used to model the decay. Total emissions (black solid lines in Fig. 5) start to rise after the first year of the analysis (1960), and initially mainly increase due to HWPs with a short life, such as bioenergy and paper. For bioenergy, which is a short-lived product, combustion releases the carbon stored in the wood to the atmosphere shortly after harvest. The situation is different for HWPs with a longer life, as they store carbon for some years after harvest, thereby gaining time for the HWP sink. Emissions from oxidation of wood used in buildings become relevant for longer timescales, representing a legacy of the HWP sector with emissions postponed up to 100 years after the end of the analysis period. For example, 50 years after the last harvest considered in this study, around 7% of the CO_2_ from the 2015 harvest remains in storage in Norway, 6% in Sweden, and 3% in Finland.

We compare the CO_2_ emissions from the actual oxidation of carbon in the HWP pool (the black lines in Fig. 5) with the common simplified assumption of instantaneous oxidation of all harvested materials at the year of harvest (red dotted lines in Fig. 5). This assumption is implicit in many climate impact assessment studies on forest management and forest-derived products. When considering the delay in C oxidation induced by the actual life of C in HWPs, the CO_2_ flows decrease of 48–110 million t CO_2_ per year in Sweden, 44–95 million t CO_2_ per year in Finland and 0.73–15 million t CO_2_ per year in Norway. This corresponds to an annual decrease of 64–91% per year in Finland, 49–96% per year in Sweden, and 6–91% per year in Norway, for the 1960–2015 period. On average, the annual decrease across the time period is 74% in Finland, 64% in Sweden, and 40% in Norway. This equates to a storage of about 13–30 million t C per year in Sweden, 12–26 million t C in Finland, and 0.2–4 million t C in Norway. The smaller difference in Norway is due to the product mix. In all three countries, emissions from HWPs tend to increase until the year 2000, and the growth is more linear in Sweden and Norway, owing to steadily growing outtake rates. Thereafter, emission rates from HWPs tend to stabilize, where the curves from the instantaneous oxidation is assumed continue to rise. Therefore, ignoring the role of delayed carbon emissions due to temporary storage in HWP can lead to significant biases in the quantification of the carbon balance of forest activities and forest-based systems.

We provide in Additional file 1: Figure S1 the historical emission inventories for fossil CO_2_, fossil CH_4_ and N_2_O due to production of material HWPs in the three countries. In Norway, higher emissions are associated with pulp production for the fossil CO_2_ and fossil CH_4_ emissions, while in Sweden paper together with pulp represent almost the same share of the total. In Finland, the largest emissions are attributed to the production of paper products. In Additional file 1: Figure S2 we provide the life-cycle emissions from bioenergy value chains and we see that the residential old wood stoves are the main driver in all three countries for the first half of the period until the contribution from the new wood stoves gradually increases.

### Negative emissions

In order to explore the potential for negative emissions of forest management for HWPs in Norway, Sweden, and Finland, we quantify the net emission balance between the time-distributed CO_2_ oxidation flows from HWPs (the black lines in Fig. 5) and the CO_2_ removal flows from the post-harvest forest regrowth in the assessment period (1960–2015). Results are shown in Table 1, in terms of instantaneous emissions (i.e., the net emissions at the respective year) and net cumulative emissions (the sum of the net emissions from 1960 up to the respective year). We find that, under continuous forest management, net instantaneous emissions reach a negative level before the end of the time period investigated. The net instantaneous CO_2_ emissions become negative in 2000 in Finland, 2009 in Sweden, and 2014 in Norway. For larger volumes (as in Sweden and Finland), the net instantaneous emissions become negative earlier than in the case of lower wood extraction levels (as in the case of Norway), and the point where net emissions turn negative is also influenced by the HWPs mix. In Finland, the first country to achieve negative emissions in this assessment period, the product mix is dominated in the early years by large shares of short-lived HWPs (e.g. paper and bioenergy). During the first decades, oxidation from short-lived products such as bioenergy and paper is the dominant factor, while biomass regrowth in the forest is still incipient. After this period, more long-lived HWPs are produced, thereby spreading the emission profile, and the CO_2_ sequestration flows become stronger, and net instantaneous emissions start to decrease.

**Table 1:** Net CO_2_ emissions for the 1960–2015 period with a breakdown on instantaneous and cumulative emissions

Net emissions [million t CO_2_]						
Year	Norway		Sweden		Finland	
	Instant	Cumulative	Instant	Cumulative	Instant	Cumulative
1960	1.4	1.4	3.2	3.2	7.8	7.8
1970	7.8	57.3	23.2	159	18.5	181
1980	8.2	140	30.3	424	17.9	364
1990	9.5	228	34.7	755	13.8	528
2000	8.2	316	22.8	1044	− 1.5	586
2010	3.4	375	− 3.4	1137	− 24.7	449
2015	− 3.8	375	− 27.9	1067	− 43.6	290
2100	0.1	− 241	0.7	− 2902	0.4	− 3468

Instantaneous emissions represent the net difference between the CO_2_ from time-distributed oxidation in HWPs and the CO_2_ from the post-harvest forest regrowth at the corresponding year. Cumulative emissions refer to the integral (sum over time) of the instantaneous emissions. The time-frame is expanded until 2100 (85 years after the last year from the analysis) in order to capture the long-lasting impacts from HWPs in the assessment period

Setting net emissions to zero at the initial year (1960) is inducing a penalty to the estimate of net emissions, because their accounting starts with high emission rates from oxidation of some HWPs (mostly bioenergy and paper) against slower CO_2_ sequestration from growing trees (growth rates are relatively slow the first couple of decades after harvest). Over time, the influence of this accounting artefact decreases. If we could extend the forest management dataset to cover the period before 1960, it is likely that net emissions would have turned to negative values earlier.

Wood outtakes from forests stops in 2015 (the last year of our dataset), and further CO_2_ flows are only caused by emissions from the depleting HWP pool and sequestration in the remaining growing trees. Negative emissions continue to increase and reach their maximum in 2040 at 14.6 million t CO_2_ per year in Norway, in 2043 at 91 million t CO_2_ per year in Finland, and in 2045 at 94.6 million t CO_2_ per year in Sweden. By 2100, net instantaneous emissions become close to zero as nearly all the carbon from HWPs is released and the carbon sink of forest regrowth saturates (under the simplified tree growth model assumed in this study). Regarding cumulative emissions, the timing for their switch to negative values occurs later, in 2045 in Norway, 2031 in Sweden, and 2020 in Finland, as positive emissions sum over time. By 2100, a cumulative total of negative emissions corresponding to 241 million t CO_2_ in Norway, 2902 million t CO_2_ in Sweden, and 3468 million t CO_2_ in Finland is achieved from HWPs produced between 1960 and 2015.

Our results show that HWPs ensure temporary instantaneous negative emissions, which last as long as there is continuous forest management and inflow of carbon to the HWPs pool. Forest management thus creates a sink with opportunities for negative emissions. This outcome is in line with conclusions from previous studies [21, 25, 31].

Limitations of this analysis concern the main assumptions described in the methodology section. Net emission estimates can be refined by considering more realistic forest models and historical management. Inclusion of trade data can give a more accurate description of the emission profiles from HWPs, and increase the robustness of our results. Future long-term assessments could also benefit from explicitly accounting for the effects of a background changing climate on forest dynamics [70], which will affect the sequestration rate of carbon and therefore the estimate of negative emissions. Especially for northern European countries, a warmer climate is expected to boost forest regrowth mainly because of higher atmospheric CO_2_ concentration and extension of the growing season, but at the same time it can favour natural disasters such as forest fires and insect outbreaks and increase heterotrophic respiration [71–73]. The net effects of these aspects are highly uncertain and case-specific [74], but are likely to impact forest management activities as in the case of the Gudrun storm in 2005, which considerably impacted both Sweden and Finland forestry sectors. The forestry sector can adapt and change management and silviculture practices (e.g., uneven‐aged forest structure). Also, life-cycle GHG emissions from HWP can be adapted to a backward-looking perspective (an example is to produce a historical version of the LCA prospective modelling tool THEMIS [75]). A detailed mass flow analysis with data harmonization that can directly link outtake volumes to final products will smooth imbalances in the system and achieve more refined estimates of net carbon fluxes. We expect such refinements would have some influence on the actual numerical findings, but do not expect general trends and conclusions to alter.

## Conclusions

The urgent need to mitigate climate change represents both a challenge and an opportunity for the forest sector worldwide. Fossil fuels can be substituted by using wood as material for energy generation, and HWPs can replace energy-intensive material products. HWPs are playing a key role for achieving climate change mitigation targets, as acknowledged by recent legislative efforts at the EU level that are considering the inclusion of the LULUCF sector for the Paris Agreement goals and explicitly request the accounting of the HWP climate mitigation potential.

In this study, we focus on Sweden and Finland, two EU countries and Norway, close partner of the EU which usually adopts and follows the EU regulations. We estimate that a total of approximately 6.6 billion m^3^ of wood were harvested between 1960 and 2015 in the 3 countries, and 638 million m^3^ (less than 10%) of this volume were harvested for energy production while 2.3 billion tonnes of solid products were manufactured. Our statistical analysis indicates that the Swedish forest sector is more stable and more independent of the financial market dynamics than the Norwegian and Finnish ones, which are more vulnerable to external conditions. There is also a relatively strong positive correlation between Sweden and Finland total harvest rates, and strong correlation between bioenergy uses in the countries as well as pine outtakes.

We also provide the CO_2_ emission profiles from oxidation of HWPs considering the individual product half-lives, and the associated emissions of GHGs. The fate of harvested wood can considerably affect the timing of greenhouse-gas emissions from forest sector, and actual emissions significantly differ from hypothetical emission profiles based on instantaneous oxidation. Assuming an instantaneous oxidation of HWPs would overestimate actual emissions of about 1.18 billion t CO_2_ (cumulative values for the three countries across the entire period of the analysis).

We provide estimates of the historical net CO_2_ emissions and C storage in the HWPs for the period 1960–2015 in Norway, Sweden, and Finland and we find that a 40 year period is sufficient to detect signs of negative emissions. We see that the temporary instantaneous negative emissions from HWPs last as long as there is continuous forest management and inflow of carbon to the HWP pool. Our results show that forest management creates a sink with opportunities for negative emissions.

Outcomes of this study will be instrumental for future assessments involving the forestry sector of Scandinavian countries, from net carbon balance analysis to climate impact studies. The historical trends can also be used to develop regression models and extrapolate future forest outtake volumes, which can work as a basis for common interdisciplinary studies on forest management and forest-derived products.

## Additional file

**Additional file 1: Table S1.** References and assumptions for Norway’s data set (harvested amounts, HWP and bioenergy). **Table S2.** References and assumptions for Sweden’s data set (harvested amounts, HWP and bioenergy). **Table S3.** References and assumptions for Finland’s data set (harvested amounts, HWP and bioenergy). **Table S4.** End Products Categories and Lifetimes. **Table S5.** Densities for HWP. **Table S6.** Rotation periods. **Table S7.** The estimated coefficients for the annual incremental rate and the standard deviation. **Table S8.** Legend of the covariance coefficients (i) for the Tables S9–S16. **Table S9.** Covariance coefficients of the historical trends of the total harvested volumes between each two countries. **Table S10.** Covariance coefficients of the historical trends of harvested volumes between each two countries with a breakdown on tree species. **Table S11.** Covariance coefficients of the historical trends of harvested volumes between each tree species class in Norway, where the first capital letter indicates the tree species (S: Spruce; P: Pine; B: Birch) while the second letter indicates the wood class (s: saw logs; p: pulpwood; e: wood for energy). **Table S12.** Covariance coefficients of the historical trends of harvested volumes between each tree species class in Sweden, where the first capital letter indicates the tree species (S: Spruce; P: Pine; B: Birch) while the second letter indicates the wood class (s: saw logs; p: pulpwood; e: wood for energy). **Table S13.** Covariance coefficients of the historical trends of harvested volumes between each tree species class in Finland, where the first capital letter indicates the tree species (S: Spruce; P: Pine; B: Birch) while the second letter indicates the wood class (s: saw logs; p: pulpwood; e: wood for energy). **Table S14.** Covariance coefficients of the historical trends of harvested volumes between Finland and Sweden with a breakdown on the tree species and class, where the first capital letter indicates the tree species (S: Spruce; P: Pine; B: Birch) while the second letter indicates the wood class (s: saw logs; p: pulpwood; e: wood for energy). **Table S15.** Covariance coefficients of the historical trends of harvested volumes between Finland and Norway with a breakdown on the tree species and class, where the first capital letter indicates the tree species (S: Spruce; P: Pine; B: Birch) while the second letter indicates the wood class (s: saw logs; p: pulpwood; e: wood for energy). **Table S16.** Covariance coefficients of the historical trends of harvested volumes between Sweden and Norway with a breakdown on the tree species and class, where the first capital letter indicates the tree species (S: Spruce; P: Pine; B: Birch) while the second letter indicates the wood class (s: saw logs; p: pulpwood; e: wood for energy). **Figure S1.** Historical emission inventories for CO_2_ fossil, CH_4_ fossil and N_2_O due to material HWPs manufacturing in Norway, Sweden and Finland. **Figure S2.** Historical emission inventories for CO_2_ fossil, CH_4_ fossil and N_2_O due to wood-based energy in Norway, Sweden and Finland. In Norway, the figures do not include emissions from district heating (most of the inputs to DH in Norway are by-products of other wood industries). The trends in Norway are very dependent on the reported removals of wood for energy.

## Abbreviations

HWP: harvested wood products
FAO: Food and Agriculture Organization of the United Nations
IPCC: The Intergovernmental Panel on Climate Change
LCA: life-cycle assessment
