# Supplementary material for: Contribution of forest wood products to negative emissions: historical comparative analysis from 1960 to 2015 in Norway, Sweden and Finland
Source: Carbon Balance Manag. 2018 Sep 4;13:12. doi: 10.1186/s13021-018-0101-9 (PMC6123331; doi:10.1186/s13021-018-0101-9)
Supplement: Supplementary file 1 — Additional file 1: Table S1. References and assumptions for Norway’s data set (harvested amounts, HWP and bioenergy). Table S2. References and assumptions for Sweden’s data set (harvested amounts, HWP and bioenergy). Table S3. References and assumptions for Finland’s data set (harvested amounts, HWP and bioenergy). Table S4. End Products Categories and Lifetimes. Table S5. Densities for HWP. Table S6. Rotation periods. Table S7. The estimated coefficients for the annual incremental rate and the standard deviation. Table S8. Legend of the covariance coefficients (i) for the Tables S9–S16. Table S9. Covariance coefficients of the historical trends of the total harvested volumes between each two countries. Table S10. Covariance coefficients of the historical trends of harvested volumes between each two countries with a breakdown on tree species. Table S11. Covariance coefficients of the historical trends of harvested volumes between each tree species class in Norway, where the first capital letter indicates the tree species (S: Spruce; P: Pine; B: Birch) while the second letter indicates the wood class (s: saw logs; p: pulpwood; e: wood for energy). Table S12. Covariance coefficients of the historical trends of harvested volumes between each tree species class in Sweden, where the first capital letter indicates the tree species (S: Spruce; P: Pine; B: Birch) while the second letter indicates the wood class (s: saw logs; p: pulpwood; e: wood for energy). Table S13. Covariance coefficients of the historical trends of harvested volumes between each tree species class in Finland, where the first capital letter indicates the tree species (S: Spruce; P: Pine; B: Birch) while the second letter indicates the wood class (s: saw logs; p: pulpwood; e: wood for energy). Table S14. Covariance coefficients of the historical trends of harvested volumes between Finland and Sweden with a breakdown on the tree species and class, where the first capital letter indicates the tree species [file 13021_2018_101_MOESM1_ESM.docx]

**Supplementary Information**

**Contribution of forest wood products to negative emissions: historical comparative analysis from 1960 to 2015 in Norway, Sweden and Finland**

Cristina-Maria Iordan^*1^, Xiangping Hu^1^, Anders Arvesen^1^, Pekka Kauppi^2^, Francesco Cherubini^1^

^1^ Industrial Ecology Programme, Department of Energy and Process Engineering, Norwegian University of Science and Technology (NTNU), Trondheim, Norway

^2^ Department of Environmental Sciences, University of Helsinki, Helsinki, Finland

^*^ Email: [cristina.m.iordan@ntnu.no](mailto:cristina.m.iordan@ntnu.no), Phone: +4745238084

Table S1: References and assumptions for Norway’s data set (harvested amounts, HWP and bioenergy)

| Category | | Reference | Assumptions |
| --- | --- | --- | --- |
| Harvested Amounts | | (Statistics Norway, 2016);  (Food and Agriculture Organization of the United Nations [FAO], 2015a) | 1960-1979 only total conifers (timber and wood for energy). We assume same ratio of pine and spruce within the total conifers as the average from 1980-1989. Wood for energy from FAO Dataset. |
| HWP | Timber | (Food and Agriculture Organization of the United Nations [FAO], 2015a) | We assume same production volumes in 1960 as in 1961. |
|  | Pulpwood | Norwegian Pulp and Paper Association - (TFB, 2015) |  |
| Bioenergy | District Heating | (Statistics Norway, 2017) | The value of TWh produced in 1983 is assumed to be fixed between 1960-1982 |
|  | Households and cabins | (Statistics Norway, 2017) | For 1960-1989 we assume the share of open fire from total households to be the same as the average from 1990 – 2015. We assume no new stoves until 1998. |
|  | Industry | (Statistics Norway, 2017) | For the period 1960 – 1989 we assume same relative shares from the total consumption of fuel as for year 1990. |
|  | Transport | (Statistics Norway, 2017) | Data available only after 2009. |

Table S2: References and assumptions for Sweden’s data set (harvested amounts, HWP and bioenergy)

| Category | Reference | Assumption |  |
| --- | --- | --- | --- |
| Harvested Amounts | (Swedish Forest Agency, 2015) |  | |
| HWP | (Food and Agriculture Organization of the United Nations [FAO], 2015a) | We assume same production amounts in 1960 as in 1961. | |
| Bioenergy | (Swedish Energy Agency, 2015) | For 1960-1989 we assume the ratio for each category from the total wood-based energy consumption equal to the category’s average ratio for the period 1990-1999. | |

Table S3: References and assumptions for Finland’s data set (harvested amounts, HWP and bioenergy)

| Category | Reference | Assumption |  |
| --- | --- | --- | --- |
| Harvested Amounts | (LUKE, 2014) |  | |
| HWP | (Food and Agriculture Organization of the United Nations [FAO], 2015a) | We assume same production amounts in 1960 as in 1961. | |
| Bioenergy | (Natural Resources Institute Finland, 2016) |  | |

Table S4: End Products Categories and Lifetimes

| Primary HWP | Finale HWP | Life time (years) |
| --- | --- | --- |
| Wood for Energy | Bioenergy | 4 |
| Graphic Paper, Tissue Paper | Paper | 4 |
| Wrapping paper, Case Materials, Carton board | Packaging Items | 9 |
| Mechanical, Chemical, Semi-chemical and Dissolving wood pulp | Pulp | 9 |
| Particle board, plywood, veneer sheets, fibreboard | Furniture (and building maintenance) | 43 |
| Sawnwood | Buildings | 140 |

Table S5: Densities for HWP

| HWP | Density ( kg/ m^3^) | Reference |
| --- | --- | --- |
| Particleboard | 650 | (Food and Agriculture Organization of the United Nations [FAO], 2015b) |
| Sawnwood ( conifers) | 440 | (UNECE, 2010) |
| Sawnwood ( non-conifers) | 550 | (UNECE, 2010) |
| Veneer sheets | 750 | (Food and Agriculture Organization of the United Nations [FAO], 2015b) |
| Plywood | 650 | (Food and Agriculture Organization of the United Nations [FAO], 2015b) |
| Fibreboard | 950 | (Food and Agriculture Organization of the United Nations [FAO], 2015b) |
| Fuelwood | 725 | (Food and Agriculture Organization of the United Nations [FAO], 2015b) |

Table S6: Rotation periods

| Tree Specie | Rotation Period (years) |
| --- | --- |
| Spruce | 100 |
| Pine | 90 |
| Birch | 60 |

Table S7: The estimated coefficients for the annual incremental rate and the standard deviation

| Country | Coefficients for the annual incremental rate | Standard deviation |
| --- | --- | --- |
| Norway | 0.029 | 0.009 |
| Sweden | 0.477 | 0.047 |
| Finland | 0.399 | 0.044 |

Table S8: Legend of the covariance coefficients (i) for the Tables S9 –S16

| i =1 | | | self-correlation |
| --- | --- | --- | --- |
| 0.7 | < i < | 0.99 | strongly positively correlated |
| 0.4 | < i < | 0.7 | positively correlated |
| 0.1 | < i < | 0.4 | slightly positively correlated |
| -0.1 | < i < | 0.1 | no correlation |
| -0.4 | < i < | -0.1 | slightly negatively correlated |
| -0.7 | < i < | -0.4 | negatively correlated |
| -1 | < i < | -0.7 | strongly negatively correlated |

Table S9: Covariance coefficients of the historical trends of the total harvested volumes between each two countries.

|  | **Norway** | **Sweden** | **Finland** |
| --- | --- | --- | --- |
| **Norway** | 1.00 | 0.33 | 0.38 |
| **Sweden** |  | 1.00 | 0.72 |
| **Finland** |  |  | 1.00 |

Table S10: Covariance coefficients of the historical trends of harvested volumes between each two countries with a breakdown on tree species

| \|  \| Norway \| \| \| \| --- \| --- \| --- \| --- \| \| Finland \| Spruce \| Pine \| Birch \| \| Spruce \| 0.23 \| 0.23 \| -0.05 \| \| Pine \| 0.41 \| 0.50 \| 0.40 \| \| Birch \| -0.11 \| 0.15 \| 0.51 \| | \|  \| Sweden \| \| \| \| --- \| --- \| --- \| --- \| \| Finland \| Spruce \| Pine \| Birch \| \| Spruce \| 0.64 \| 0.66 \| -0.64 \| \| Pine \| 0.69 \| 0.79 \| -0.31 \| \| Birch \| 0.14 \| 0.21 \| 0.31 \| | \|  \| Norway \| \| \| \| --- \| --- \| --- \| --- \| \| Sweden \| Spruce \| Pine \| Birch \| \| Spruce \| 0.18 \| 0.40 \| 0.20 \| \| Pine \| 0.21 \| 0.45 \| 0.29 \| \| Birch \| 0.27 \| 0.31 \| 0.41 \| |
| --- | --- | --- | --- | --- | --- | --- | --- | --- | --- | --- | --- | --- | --- | --- | --- | --- | --- | --- | --- | --- | --- | --- | --- | --- | --- | --- | --- | --- | --- | --- | --- | --- | --- | --- | --- | --- | --- | --- | --- | --- | --- | --- | --- | --- | --- | --- | --- | --- | --- | --- | --- | --- | --- | --- | --- | --- | --- | --- | --- | --- | --- | --- |

Table S11: Covariance coefficients of the historical trends of harvested volumes between each tree species class in Norway, where the first capital letter indicates the tree species (S – Spruce, P-Pine, B -Birch) while the second letter indicates the wood class (s – saw logs, p – pulpwood, e – wood for energy).

| **Norway** | **Ss** | **Sp** | **Se** | **Ps** | **Pp** | **Pe** | **Bs** | **Bp** | **Be** |
| --- | --- | --- | --- | --- | --- | --- | --- | --- | --- |
| **Ss** | **1.00** | **0.37** | **-0.18** | **0.58** | **0.42** | **-0.26** | **-0.16** | **0.29** | **-0.05** |
| **Sp** |  | **1.00** | **0.02** | **0.65** | **0.78** | **0.04** | **-0.23** | **-0.14** | **0.25** |
| **Se** |  |  | **1.00** | **0.16** | **0.12** | **0.99** | **-0.34** | **-0.33** | **0.88** |
| **Ps** |  |  |  | **1.00** | **0.61** | **0.18** | **-0.47** | **-0.26** | **0.35** |
| **Pp** |  |  |  |  | **1.00** | **0.12** | **-0.14** | **0.11** | **0.33** |
| **Pe** |  |  |  |  |  | **1.00** | **-0.40** | **-0.43** | **0.89** |
| **Bs** |  |  |  |  |  |  | **1.00** | **0.81** | **-0.56** |
| **Bp** |  |  |  |  |  |  |  | **1.00** | **-0.46** |
| **Be** |  |  |  |  |  |  |  |  | **1.00** |

Table S12: Covariance coefficients of the historical trends of harvested volumes between each tree species class in Sweden, where the first capital letter indicates the tree species (S – Spruce, P-Pine, B -Birch) while the second letter indicates the wood class (s – saw logs, p – pulpwood, e – wood for energy).

| **Sweden** | **Ss** | **Sp** | **Se** | **Ps** | **Pp** | **Pe** | **Bs** | **Bp** | **Be** |
| --- | --- | --- | --- | --- | --- | --- | --- | --- | --- |
| **Ss** | **1.00** | **0.53** | **0.57** | **0.95** | **0.57** | **0.57** | **0.35** | **-0.44** | **0.07** |
| **Sp** |  | **1.00** | **0.27** | **0.48** | **0.86** | **0.26** | **-0.21** | **0.32** | **0.14** |
| **Se** |  |  | **1.00** | **0.63** | **0.44** | **0.98** | **0.37** | **-0.56** | **0.70** |
| **Ps** |  |  |  | **1.00** | **0.69** | **0.69** | **0.34** | **-0.41** | **0.18** |
| **Pp** |  |  |  |  | **1.00** | **0.52** | **-0.12** | **0.22** | **0.30** |
| **Pe** |  |  |  |  |  | **1.00** | **0.35** | **-0.52** | **0.69** |
| **Bs** |  |  |  |  |  |  | **1.00** | **-0.54** | **0.06** |
| **Bp** |  |  |  |  |  |  |  | **1.00** | **0.03** |
| **Be** |  |  |  |  |  |  |  |  | **1.00** |

Table S13: Covariance coefficients of the historical trends of harvested volumes between each tree species class in Finland, where the first capital letter indicates the tree species (S – Spruce, P-Pine, B -Birch) while the second letter indicates the wood class (s – saw logs, p – pulpwood, e – wood for energy).

| **Finland** | Ss | Sp | Se | Ps | Pp | Pe | Bs | Bp | Be |
| --- | --- | --- | --- | --- | --- | --- | --- | --- | --- |
| Ss | 1.00 | 0.09 | 0.10 | 0.65 | **0.80** | 0.13 | -0.61 | **0.72** | -0.67 |
| Sp |  | 1.00 | 0.03 | 0.34 | -0.04 | -0.09 | 0.39 | -0.15 | 0.30 |
| Se |  |  | 1.00 | 0.11 | 0.40 | **0.95** | -0.38 | 0.15 | 0.49 |
| Ps |  |  |  | 1.00 | 0.47 | 0.08 | -0.03 | 0.41 | -0.31 |
| Pp |  |  |  |  | 1.00 | 0.49 | -0.65 | **0.88** | -0.46 |
| Pe |  |  |  |  |  | 1.00 | -0.43 | 0.30 | 0.40 |
| Bs |  |  |  |  |  |  | 1.00 | -0.50 | 0.42 |
| Bp |  |  |  |  |  |  |  | 1.00 | -0.58 |
| Be |  |  |  |  |  |  |  |  | 1.00 |

Table S14: Covariance coefficients of the historical trends of harvested volumes from Finland and Sweden with a breakdown on the tree species and class, where the first capital letter indicates the tree species (S – Spruce, P-Pine, B -Birch) while the second letter indicates the wood class (s – saw logs, p – pulpwood, e – wood for energy).

|  | **Sweden** | | | | | | | | | |
| --- | --- | --- | --- | --- | --- | --- | --- | --- | --- | --- |
| **Finland** | Ss | Sp | Sf | Ps | Pp | Pf | Bs | Bp | Bf |  |
| Ss | **0.76** | **0.08** | **0.63** | **0.77** | **0.24** | **0.65** | **0.68** | **-0.74** | **0.07** |  |
| Sp | **0.00** | **0.10** | **0.16** | **-0.01** | **0.05** | **0.14** | **-0.02** | **-0.17** | **-0.07** |  |
| Sf | **0.18** | **0.24** | **0.67** | **0.29** | **0.41** | **0.68** | **-0.21** | **-0.10** | **0.76** |  |
| Ps | **0.47** | **0.01** | **0.41** | **0.54** | **0.23** | **0.46** | **0.24** | **-0.45** | **0.06** |  |
| Pp | **0.70** | **0.32** | **0.77** | **0.76** | **0.52** | **0.80** | **0.49** | **-0.42** | **0.47** |  |
| Pf | **0.22** | **0.28** | **0.64** | **0.33** | **0.46** | **0.67** | **-0.16** | **-0.01** | **0.80** |  |
| Bs | **-0.57** | **-0.18** | **-0.52** | **-0.57** | **-0.27** | **-0.52** | **-0.43** | **0.44** | **-0.22** |  |
| Bp | **0.67** | **0.31** | **0.59** | **0.72** | **0.47** | **0.62** | **0.54** | **-0.29** | **0.36** |  |
| Bf | **-0.48** | **0.07** | **-0.05** | **-0.44** | **0.01** | **-0.08** | **-0.73** | **0.37** | **0.30** |  |

Table S15: Covariance coefficients of the historical trends of harvested volumes from Finland and Norway with a breakdown on the tree species and class, where the first capital letter indicates the tree species (S – Spruce, P-Pine, B -Birch) while the second letter indicates the wood class (s – saw logs, p – pulpwood, e – wood for energy).

|  | **Norway** | | | | | | | | |
| --- | --- | --- | --- | --- | --- | --- | --- | --- | --- |
| **Finland** | Ss | Sp | Sf | Ps | Pp | Pf | Bs | Bp | Bf |
| Ss | **0.14** | **0.41** | **0.00** | **0.46** | **0.09** | **0.06** | **-0.71** | **-0.75** | **0.19** |
| Sp | **-0.18** | **0.00** | **-0.07** | **-0.14** | **-0.22** | **-0.07** | **0.33** | **0.02** | **-0.20** |
| Sf | **-0.24** | **0.03** | **0.84** | **0.18** | **0.02** | **0.84** | **-0.39** | **-0.48** | **0.70** |
| Ps | **0.21** | **0.01** | **0.09** | **0.25** | **-0.16** | **0.12** | **-0.44** | **-0.43** | **0.16** |
| Pp | **0.13** | **0.53** | **0.37** | **0.57** | **0.32** | **0.42** | **-0.82** | **-0.78** | **0.61** |
| Pf | **-0.09** | **0.15** | **0.79** | **0.32** | **0.17** | **0.78** | **-0.47** | **-0.47** | **0.74** |
| Bs | **0.01** | **-0.46** | **-0.27** | **-0.46** | **-0.28** | **-0.33** | **0.67** | **0.68** | **-0.40** |
| Bp | **0.21** | **0.53** | **0.17** | **0.56** | **0.40** | **0.24** | **-0.76** | **-0.62** | **0.51** |
| Bf | **-0.43** | **-0.41** | **0.40** | **-0.39** | **-0.25** | **0.36** | **0.49** | **0.27** | **0.09** |

Table S16: Covariance coefficients of the historical trends of harvested volumes from Sweden and Norway with a breakdown on the tree species and class, where the first capital letter indicates the tree species (S – Spruce, P-Pine, B -Birch) while the second letter indicates the wood class (s – saw logs, p – pulpwood, e – wood for energy).

|  | **Norway** | | | | | | | | |
| --- | --- | --- | --- | --- | --- | --- | --- | --- | --- |
| **Sweden** | Ss | Sp | Sf | Ps | Pp | Pf | Bs | Bp | Bf |
| Ss | **0.02** | **0.37** | **0.09** | **0.55** | **0.11** | **0.18** | **-0.66** | **-0.73** | **0.29** |
| Sp | **-0.19** | **0.24** | **0.25** | **0.29** | **0.15** | **0.33** | **-0.22** | **-0.32** | **0.39** |
| Sf | **-0.22** | **0.21** | **0.59** | **0.32** | **0.04** | **0.64** | **-0.67** | **-0.80** | **0.64** |
| Ps | **0.06** | **0.30** | **0.20** | **0.57** | **0.12** | **0.28** | **-0.75** | **-0.79** | **0.38** |
| Pp | **-0.10** | **0.17** | **0.41** | **0.39** | **0.18** | **0.47** | **-0.48** | **-0.51** | **0.55** |
| Pf | **-0.17** | **0.19** | **0.60** | **0.36** | **0.06** | **0.65** | **-0.72** | **-0.82** | **0.65** |
| Bs | **0.25** | **0.42** | **-0.24** | **0.34** | **0.25** | **-0.22** | **-0.42** | **-0.30** | **-0.03** |
| Bp | **0.19** | **0.01** | **0.03** | **-0.03** | **0.32** | **-0.01** | **0.42** | **0.63** | **0.05** |
| Bf | **-0.01** | **0.20** | **0.78** | **0.27** | **0.31** | **0.75** | **-0.40** | **-0.28** | **0.75** |

| 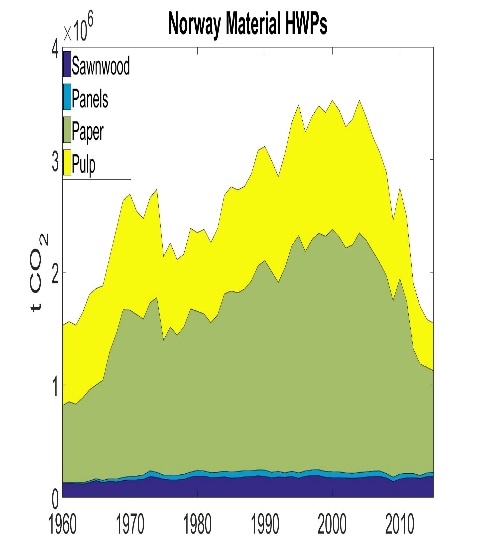 | 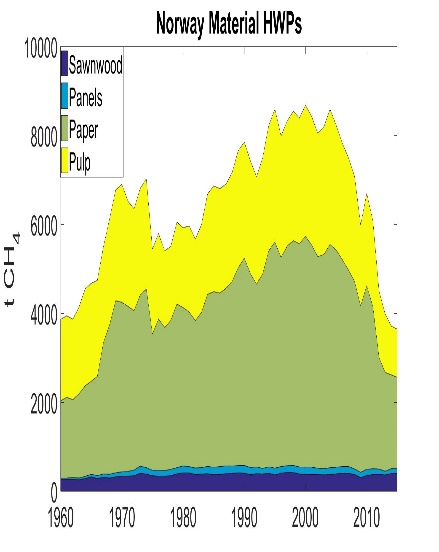 | 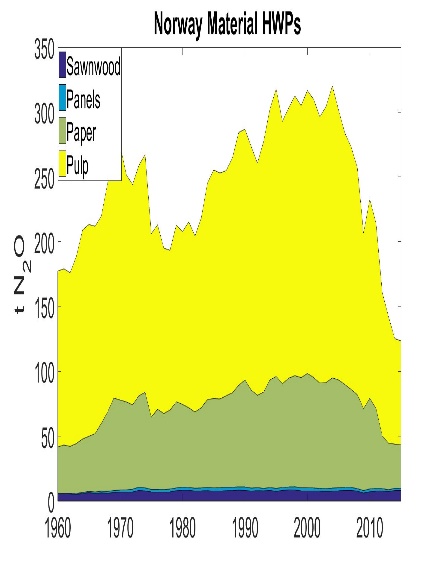 |
| --- | --- | --- |
| 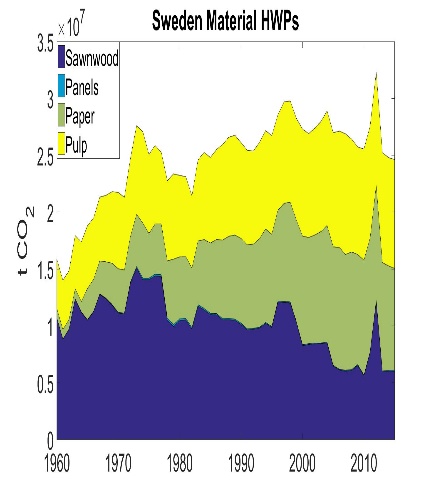 | 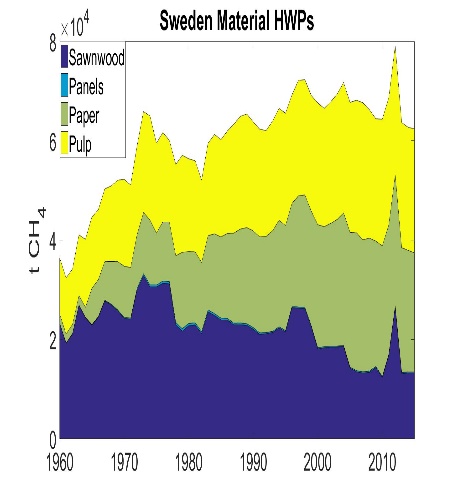 | 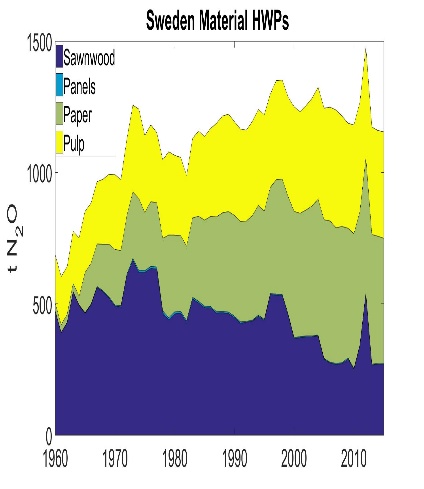 |
| 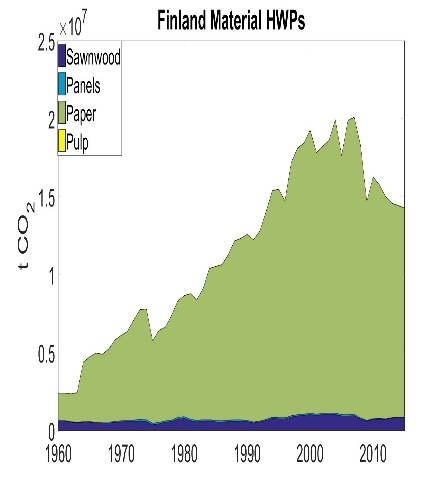 | 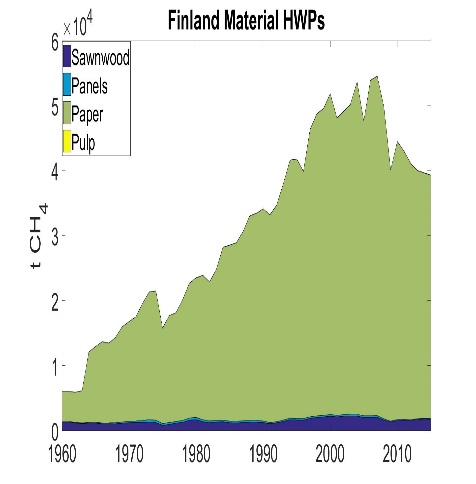 | 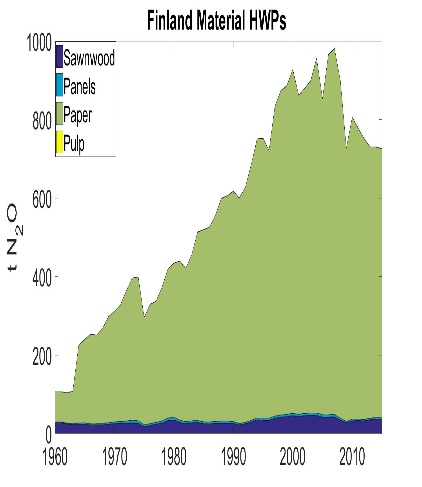 |

Figure S1: Historical emission inventories for CO_2_ fossil, CH_4_ fossil and N_2_O due to material HWP in Norway, Sweden and Finland.

| 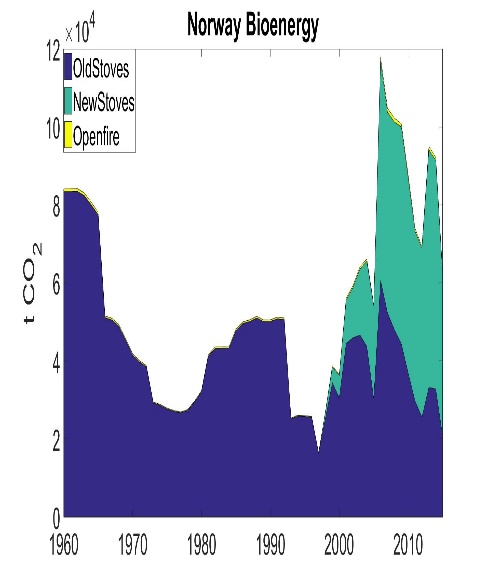 | 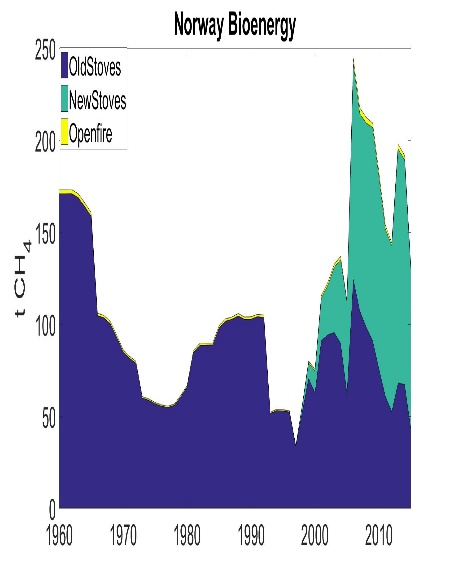 | 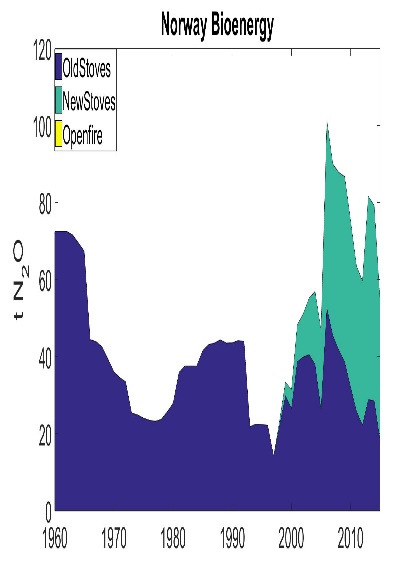 |
| --- | --- | --- |
| 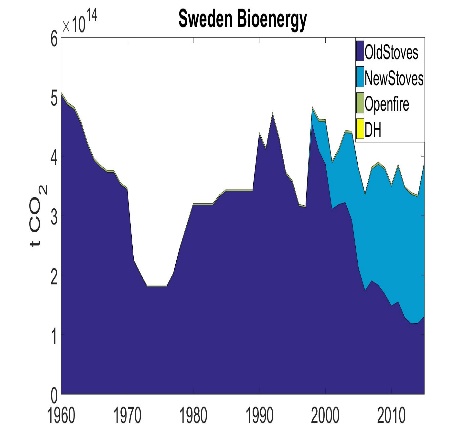 | 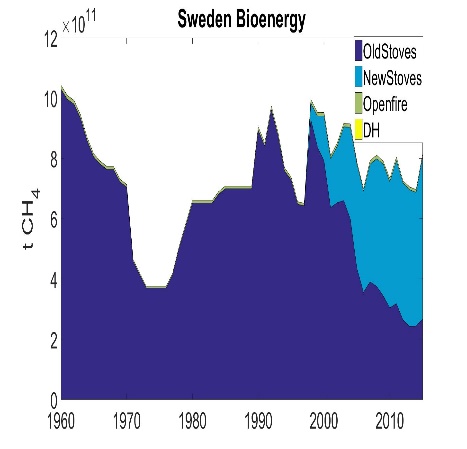 | 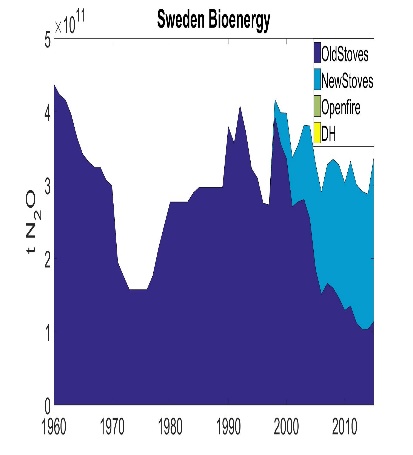 |
| 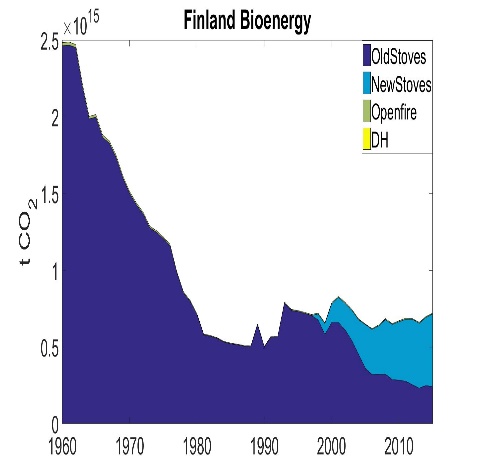 | 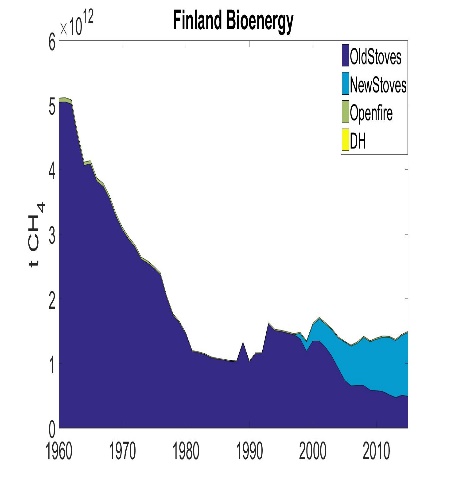 | 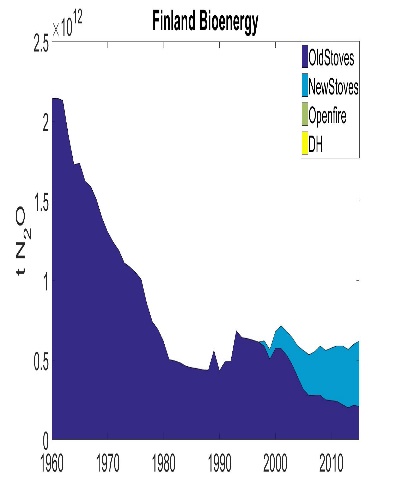 |

Figure S2: Historical emission inventories for CO_2_ fossil, CH_4_ fossil and N_2_O due to wood-based energy in Norway, Sweden and Finland. In Norway, the figures do not include emissions from district heating (most of the inputs to DH in Norway are by-products of other wood industries). The trends in Norway are very dependent on the reported removals of wood for energy.

**References:**

Food and Agriculture Organization of the United Nations [FAO], (2015a) FAOSTAT. Forestry Production and Trade 1960 - 2015 <http://www.fao.org/faostat/en/#data/FO>.

Food and Agriculture Organization of the United Nations [FAO], (2015b) Global Forest Resources Assessment 2015: How are the world’s forests changeing? , Rome, Italy.

LUKE, (2014) Statistical Yearbook of Forestry 2014; Official statistics of Finland. Finnish Forest Research Institute.

Natural Resources Institute Finland, (2016) Wood in energy generation, <http://stat.luke.fi/puun-energiakaytto>.

Statistics Norway, (2016) The National Forest Inventory 2011-2015 - Commercial roundwood removals by species of tree, Oslo.

Statistics Norway, (2017) Energy and manufacturing: Energy 30.07.2017 ed, <https://www.ssb.no/en/energi-og-industri/statistikker/fjernvarme>.

Swedish Energy Agency (2015) Energy in Sweden 2015.

Swedish Forest Agency, (2015) Statistical Database on Forestry

TFB, (2015) Norwegian Pulp and Paper Association - Key Figures 2015 <https://www.norskindustri.no/contentassets/10b0b5431ab24b46aa94602a71f6bd1d/nokkeltall-for-treforedlingsbransjen-2016.pdf>.

UNECE, (2010) United Nations Economic Commision for Europe. Forest product conversion factors for the UNECE region <https://www.unece.org/fileadmin/DAM/timber/publications/DP-49.pdf>.
